# The Association between a Minimum Amount of Physical Activity and Subsequent Muscle Strength and Balance in Older Adults: A Prospective Study

**DOI:** 10.3390/bs13040316

**Published:** 2023-04-06

**Authors:** Yuan-Yu Li, Ming-Chun Hsueh, Jong-Hwan Park, Ting-Fu Lai, Yi-Chuan Hung, Yung Liao

**Affiliations:** 1 Department of Health Promotion and Health Education, National Taiwan Normal University, 162, Heping East Road Section 1, Taipei 106, Taiwan; 2 Graduate Institute of Sport Pedagogy, University of Taipei, No. 101, Sec. 2, Jhongcheng Rd., Shilin Dist., Taipei 11153, Taiwan; 3 Health Convergence Medicine Research Group, Biomedical Research Institute, Pusan National University Hospital, 179, Gudeok-Ro, Seo-Gu, Busan 49241, Republic of Korea; 4 Department of Sport Management, National Taiwan University of Sport, 16, Sec. 1, Shuang-Shih Rd., Taichung 404, Taiwan; 5 Graduate Institute of Sports, Leisure and Hospitality Management, National Taiwan Normal University, 162, Heping East Road Section 1, Taipei 106, Taiwan

**Keywords:** MVPA, older adults, physical function

## Abstract

This study aimed to investigate the association between 15 min of daily moderate-to-vigorous physical activity (MVPA) and subsequent muscle strength and balance in older adults. Data concerning community-dwelling older Taiwanese adults (mean age, 69.5 years) were collected during the baseline period (2018) and at a 12-month follow-up survey (2019). Time spent performing MVPA was objectively assessed using a triaxial accelerometer (ActiGraph wGT3x-BT) at baseline. Upper and lower limb muscle strength were measured using handgrip strength and a five-times sit-to-stand test, respectively. Balance was assessed using a one-leg standing test. The 12-month variations in muscle strength and balance were calculated through subtracting follow-up data from baseline data. A forced entry-adjusted logistic regression analysis was conducted. In total, 65.2% of participants engaged in at least 15 min of daily MVPA in the baseline survey. After adjusting for confounders, older adults who achieved 15 min of daily MVPA during the baseline period were more likely to maintain or improve balance performance (odds ratio, 8.12). Performing 15 min of daily MVPA was found to benefit older adults’ subsequent balance performance but not muscle strength.

## 1. Introduction

Worldwide, humans are living longer than ever before. Global Health Observatory data indicate that the global average life expectancy in 2016 was 72 years [1]. However, longer life expectancies have led to a rapidly increasing proportion of older adults. Older adults accounted for 9% of the global population in 2018, and this proportion is projected to increase to 16% by 2050 [2]. In Taiwan, older adults accounted for 14.05% of the country’s total population in 2018 and this proportion is predicted to reach 36.5% by 2050 [3]. While older adults presently have greater longevity than before, ill health is likely to lead to increasingly higher health expenditure and government funding to meet healthcare needs [4,5]. Therefore, a key World Health Organization goal for rapidly aging societies is “healthy aging”, to maintain functional ability to enable wellbeing in older adults [6]. In particular, physical functional ability is essential for older adults to achieve personal mobility required for performing activities of daily living safely and independently [7,8]. However, physical functional ability declines with age [9] and age-related decreases in physical function are associated with higher risks of mortality and disability in older adult populations [10,11]. Therefore, maintaining and even improving physical function later in life is critical for healthy aging.

Ensuring physical function in older adults involves improving or maintaining muscle strength to support optimal functional performance and independence in later life [12,13,14], given skeletal muscles are responsible for all voluntary movements [15]. Reduced muscle strength has been found to be associated with an increased risk of falls, hospitalization, and poor quality of life in older adults [16,17]. In addition to muscle strength, balance is a fundamental requirement for independent functioning in older adults [18]. Impaired balance is related to an increased risk of falls, leading to serious injury, with higher levels of morbidity, hospitalization, and mortality [19,20]. Given progressively decreasing muscle strength and balance with aging [21], there is an urgent need to identify lifestyle factors related to muscle strength and balance to design effective behavioral change strategies for older populations.

Physical activity (PA) is well documented as facilitating the improvement and maintenance of physical function in older adults and is essential in postponing morbidity associated with aging [22,23,24,25]. According to current PA recommendations for older populations, older adults should engage in at least 150 min of moderate-to-vigorous PA (MVPA) or 75 min of vigorous-intensity PA (VPA) per week to obtain substantial health benefits [26]. These recommended levels of PA have been reported to result in a positive association between muscle strength and balance in older adults [27,28]. While substantial benefits of meeting PA recommendations have been reported, the percentage of older adults who meet PA guidelines remains low in the United States [29] and in certain East Asian countries [30,31]. Meeting the current PA guidelines (150 min/week of MVPA) may be especially challenging for such adults, and a smaller amount of MVPA might be easier to achieve, particularly in older adult populations experiencing a decline in physical function. Although previous intervention studies have examined PA programs for increasing balance and muscle strength [32,33], few studies have examined the effects of MVPA in terms of its duration. Two large cohort studies reported that achieving a minimum amount of PA (15 min/day or 90 min/week) was sufficient to show an association between that minimum amount of PA and a reduced risk of cancer and all-cause mortality as well as an extended life expectancy [34,35]. Therefore, an investigation is needed to determine whether this minimum amount of PA, which may be easier for older adults to adhere to and achieve, could help older adults maintain or improve their muscular and balance functions. This study aimed to investigate the association between a minimum amount of PA (15 min/day) and 12-month changes in muscle strength and balance performance among community-dwelling older adults.

## 2. Materials and Methods

### 2.1. Participants

The study participants were comprised of community-dwelling older adults from 28 neighborhoods in the Taipei metropolitan area (including Taipei City and New Taipei City) in Taiwan who were able to walk independently. In the Taipei metropolitan area, 29.1% of the population are aged >65 years, comprising the highest population of older adults among all cities in Taiwan (approximately 1,020,000 individuals). Therefore, older adults living in the Taipei metropolitan area were targeted for inclusion in this study. We collected baseline data from April to September 2018 and follow-up data 12 months later, from April to September 2019. Baseline data collection included: (i) a self-administered questionnaire (personal information, health behavior, and health status), (ii) a performance-based physical function test (handgrip, one-leg standing test, and five sit-to-stand tests), and (iii) an accelerometer-assessed activity (accelerometer worn for seven days consecutively). Performance-based physical function tests were conducted during the follow-up survey.

**Figure 1:**
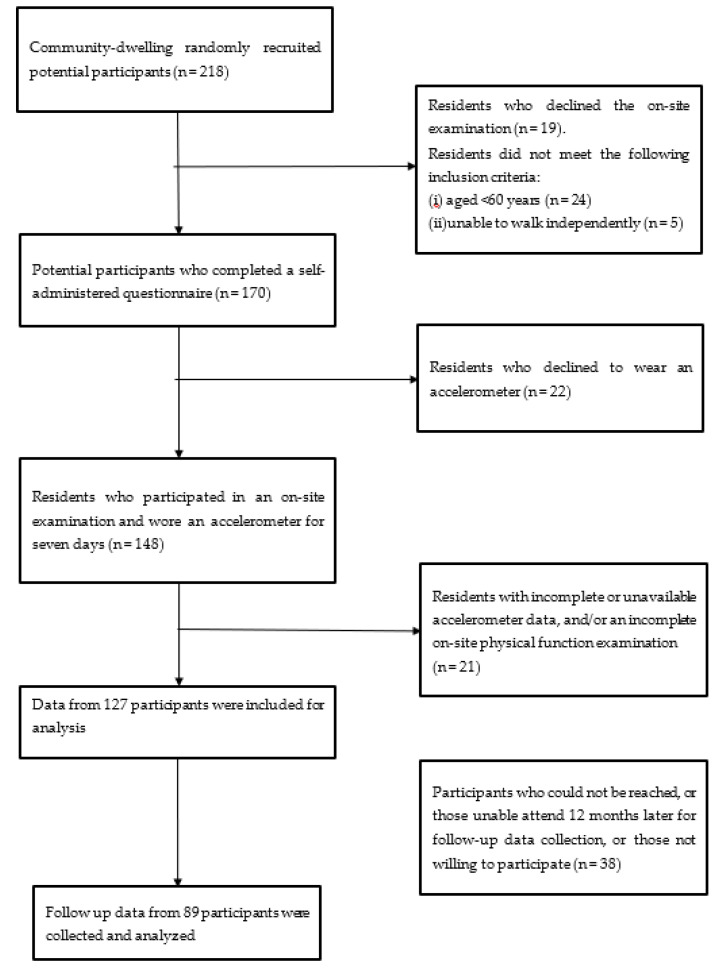
Study float chart.

In total, 218 potential participants were randomly recruited using convenience sampling. Of these, 19 declined to attend the on-site examination and 29 did not meet the inclusion criteria for age (*n* = 24) and independent walking ability (*n* = 5). A total of 170 participants completed self-administered questionnaires, of whom, 148 completed an on-site examination and wore an accelerometer for seven days. After data cleaning, 127 participants provided complete data for analysis. After our follow-up estimation 12 months later, participants who could not be reached, who were unable to attend, or who were unwilling to attend were excluded (*n* = 38). Complete data concerning 89 participants were included in this study. No recommendation was given to the participants in terms of PA after baseline, and no PA was registered at follow-up. A flowchart of the participant recruitment process is shown in Figure 1. Each participant provided written informed consent prior to study participation. This study was supported by the Ministry of Science and Technology of Taiwan (Grant number: MOST 106-2410-H-003-144-MY2). Ethical approval was obtained from the university’s research ethics committee (REC number: 201711HM003).

### 2.2. Objectively Measured MVPA

Sensitive accelerometers such as ActiGraph GT3X detect human micromovements accurately and provide objectively measured MVPA counts (ActiGraph Corporation, Pensacola, FL, USA). The validity and reliability of triaxial accelerometer models for estimating PA have previously been reported [36], and our data were collected using standard methods. All data analyses employed a 1-s versus 60-s epoch length. Participants were requested to wear an accelerometer with an elasticized belt around the waist for seven consecutive days, except when bathing (to avoid any water contact). Three movement axes were recorded for each participant. Accelerometer data were considered valid if the device was worn for ≥10 h each day for at least four consecutive days (excluding the day they received it). Non-wear time was removed during data processing (defined as 60 consecutive min of zero count, allowing interruption periods of 2 min) [37]. MVPA was defined as all activity ≥2020 counts per min [38]. ActiLife v 6.0 software (Pensacola, FL, USA) was used for accelerometer data analysis. Based on a previous study, participants were divided into two groups, namely, those reaching the minimum amount of MVPA (15 min per day) and those who did not [35].

### 2.3. Muscle Strength

Muscle strength was measured using two physical function tests, namely, a handgrip strength test for upper limb strength and a five-times sit-to-stand test (STS) for lower body strength.

(a) Upper limb strength (hand grip strength test)
  To estimate upper limb strength, a Jamar Plus+ Digital Hand Dynamometer was used to assessed handgrip strength. This hand grip strength tool is a validated instrument for obtaining rapid results concerning an individual’s general muscle strength [39]. We conducted tests using this tool on both the dominant and non-dominant hands three times, with a timed rest break between each test. Participants were instructed to stand in a natural position and hold the dynamometer in the dominant hand and with the elbow flexed to 90°, while the arm of the non-tested hand rested alongside the body. To ensure a comfortable palm fit on the dynamometer, the width of the dynamometer was adjusted according to the size of the hand. Participants were requested to apply the strongest and fastest possible pressure to the probe and their best test score was used in the analysis [40].
(b) Lower limb strength (five-times STS tests)
  To estimate lower body strength, we used the five-times STS test [41], which is a functional strength measurement that approximates body movement in daily life and has been widely used in previous studies to measure of lower limb strength [42,43]. Participants were asked to rise from an armless chair five times as fast as possible with their arms folded. The time began on the command of “go” and stopped when the participant completed five repetitions. Each participant was allowed to practice before the timing of three trials, and the average rate from three trials was used in the subsequent analysis.

### 2.4. Balance

A one-leg standing test was used to estimate balance in older adults. One-leg balance performance is a simple and easily conducted predictor of harmful falls and can be easily incorporated into the comprehensive functional evaluation of older adults [44]. To perform the test, all participants chose one dominant or stable leg and lifted the opposite leg to a comfortable height without holding on to a supportive structure. Participants were asked to look straight ahead with their arms at their sides, with no specific regulation on how high the leg should be lifted. The researchers supervised the participants during the estimation, and the process ended when: (i) a participant’s foot touched the floor in <60 s, (ii) a participant maintained this posture for >60 s, and (iii) a participant’s arms touched something for support. The maximum time for each participant’s leg was recorded, and scores ≥60 s were recorded as 60 s.

We calculated the 12-month variations in muscle strength and balance through subtracting follow-up data from baseline strength and balance data. When a value was zero or positive, it was categorized as maintenance/improvement in muscle strength and balance. When a value was negative, it was categorized as a ‘decline’ in muscle strength and balance.

### 2.5. Covariates

The covariates in this study included age, sex, education level, marital status, employment, living status, health behavior (smoking history, alcohol consumption, healthy diet), self-reported health status, the presence of certain comorbidities (hypertension, hyperlipidemia, diabetes, and depression), body mass index (BMI, kg/m^2^), accelerometer wear time, and sedentary time (>100 counts/min) [38,45].

### 2.6. Statistical Analyses

Complete data from 89 participants constituted the data for all analyses. Chi-square tests were performed to compare the proportional differences between the two participant groups (those reaching 15 min of MVPA per day and those who did not). Logistic regression analysis was used to determine the association between achieving 15 min of objectively measured MVPA per day and changes in muscle strength and balance. Additionally, forced entry-adjusted logistic regression was conducted to estimate the odds ratios (ORs) of maintaining and improving muscle strength and balance according to the definition of achieving 15 min of MVPA from baseline data. Model 1 was not adjusted for. After adjusting for sociodemographic characteristics (yielding Model 2), other potential confounders were further adjusted for in terms of sedentary behavior and accelerometer wear time (Model 3). Adjusted ORs and 95% confidence intervals (CIs) were calculated for each variable. All statistical analyses were conducted using IBM SPSS (version 23.0; IBM, Chicago, IL, USA) software, and the level of significance was set at *p* < 0.05.

## 3. Results

Table 1 shows the participants’ sociodemographic characteristics. Eighty-nine older adults were included in our study (mean age, 69.5 years; standard deviation [SD] 4.88). Older adults who were able to perform 15 min of daily MVPA tended to be female (62.1%), had at least a high school degree (70.7%), were married (79.3%), did not have a full-time job (98.3%), and were not depressed (93.1%).

Table 2 presents the results of descriptive statistics used to objectively assess PA and upper/lower limb muscle strength and balance in the baseline and follow-up measurements. Of all participants, the average wear time (min/day) was 921.86 (SD 84.99) min, with 291.60 (SD 79.40) min of light-intensity PA time, and 25.92 (SD 22.63) min of cumulative MVPA.

Table 3 shows percentages in terms of maintenance, improvement, or decline in physical function. In general, >50% of participants had retained their measures at follow-up.

Logistic regression analyses were performed to investigate the association between achieving 15 min/day of exercise and subsequent muscle strength and balance, with the results shown in Table 4. There was a significant association between participants who achieved the daily 15 min MVPA and the five-times STS and one-leg standing tests (OR 4.644, 95% CI 1.80–11.96, *p* = 0.001; OR 6.000, 95% CI 2.08–17.32, *p* = 0.001; respectively, Model 1). After adjusting for socio-demographic variables (Model 2), only the one-leg standing test correlated with the 15 min MVPA (OR = 6.46, 95% CI = 1.46–28.7, *p*-value = 0.014); the STS was no longer significant. Furthermore, the one-leg standing test remained positively associated with the minimum MVPA after adjusting for other potential confounders (OR 8.12, 95% CI 1.61–40.97, *p* = 0.011; Model 3).

## 4. Discussion

This prospective study aimed to investigate the association between a minimum amount of MVPA (15 min/day) and the subsequent performance of older adults in terms of muscle strength and balance over a 12-month follow-up period. Our findings indicated that older adults who engaged in 15 min of daily MVPA in the baseline phase were more likely to maintain or improve their standing balance than those who did not. These novel findings add further support to Wen et al.’s finding that a minimum amount of MVPA had health benefits in relation to non-communicable diseases and mortality [35]. Our findings also indicated that this minimum amount of daily MVPA can also be beneficial for balance in older adults over a long-term period. Therefore, our results suggest that including a 15 min daily MVPA intervention program within the current PA guidelines (at least 30 min of MVPA/day) [29,46] may support older adults in terms of better muscular function and should be considered for the prevention of functional decline.

Balance concerns the ability to maintain equilibrium when moving or stationary [29], which involves controlling posture while standing or walking, or anticipating body movements, i.e., integrating several human body systems. Balance is an important core physical function, particularly in older adults, owing to their high risk of falls [29,47]. PA has been shown to have positive effects on functional ability, co-ordination, and balance (postural stability) [48]. One cross-sectional study found that, with an increase in MVPA and regardless of its intensity, older adults were more likely to have better functional balance, which is consistent with our follow-up study [49]. Furthermore, although a previous meta-analysis and systematic review reported that older adults who participated in an exercise program (3 h/week) had improved balance [50], our study found that engaging in only 15 min of daily MVPA (1.5 h/week) was also positively associated with subsequent improved balance in older adults. However, it remains unclear why 15 min of daily MVPA contributes to long-term benefits for older adults in terms of balance. The dose-response relationship between PA and health benefits may explain this benefit, in that substantial health benefits have been shown for older adults undertaking a small amount of PA compared with inactive individuals [51]. Several studies have reported dose-response relationships between PA and breast cancer, lung cancer, coronary heart disease, type 2 diabetes, and hypertension [52]. Our findings also suggest that older adults undertaking a minimum dose of 15 min of daily MVPA could have substantial benefits for long-term body balance compared with less active older adults. We also noted that our study participants were older adults facing physical functional decline with aging; hence, it is possible that this minimum PA could have more profound effects in the older adult population. Further prospective or experimental studies are warranted to confirm these findings. Our study sample mostly comprised female participants; therefore, these findings could facilitate the design of effective programs for older women.

In our study, neither upper nor lower limb strength was associated with the minimum amount of daily MVPA. A previous cross-sectional study showed that older adults who engaged in just five minutes of daily MVPA had a better performance in handgrip and five-time STS tests [53], which is inconsistent with our previous findings. However, the reason for this inconsistency remains unclear and requires further investigation. Possible explanations may be as follows. First, the advanced American College of Sports Medicine Guidelines for Exercise Testing and Prescription (2014) employ a frequency, intensity, time, and type formula [54]. The accelerometer used in our study can accurately estimate the intensity, frequency, and time of PA but not the type of MVPA. In terms of moderate-to-vigorous-intensity aerobic PA (such as brisk walking, yoga, cycling, dancing, and swimming), older adults engaging in a variety of MVPA types may use different muscle groups that have different effects on their physical function. Second, the PA guidelines for older adults recommend that older adults should engage in both at least 150 min of aerobic MVPA and muscle-strengthening activities that involve all major muscle groups twice a week to obtain various health benefits [29]. However, in the present study, we used an accelerometer to determine participants’ MVPA, which was limited to obtaining information on muscle-strengthening activities. Therefore, to further understand the prospective association between minimum MVPA and muscle strength, it is critical to use both objective and subjective measurements (i.e., a valid questionnaire) to examine this issue.

Our study had several strengths. Accelerometer-assessed PA data provided accurate evidence of daily MVPA accumulation among older adults. Moreover, our 12-month follow-up evaluation ensured that the outcome variables were measured twice, which provided stronger evidence for a cause-and-effect relationship between the minimum amount of MVPA and changes in physical function among older adults. Nevertheless, our study had several limitations. Although an accelerometer can detect older adults’ micro-movements accurately and provide objective intensity and activity counts, the types of PA performed (i.e., walking, yoga, or muscle-strengthening activities) were not identifiable. Moreover, owing to convenience sampling of community-dwelling older adults in Taipei City only, our results are not readily generalizable. Regarding the recruitment procedure, healthy older adults were more likely to participate; hence, our findings should be interpreted with caution. We were able to conclude that an objectively measured minimum amount of MVPA tended to maintain and even improve subsequent balance in older adults. However, this minimum amount was not found to be related to subsequent muscle strength in our study. Nevertheless, our study suggests that any PA intervention or fundamental exercise prescription aimed at balance or falls prevention in older adults should incorporate at least 15 min of PA/day.

Furthermore, when health promoters or policymakers develop strategies to prevent functional decline in an aging population, the recommended minimum amount of MVPA should be considered.

## 5. Conclusions

Fifteen minutes of daily MVPA was found to be beneficial for older adults’ subsequent balance performance, but not beneficial in terms of improving muscle strength.

## Figures and Tables

**Table 1:** Participant characteristics.

Variables	Total Sample (N = 89)	MVPA < 15 min/day (N = 31)	MVPA ≥ 15 min/day (N = 58)	*p* (<0.05)
Baseline-Age (M ± SD)	69.54 ± 4.88	71.94 ± 5.35	68.26 ± 4.11	0.012 *
Ages 65–74	84.3%	71.0%	91.4%	
Aged ≥75	15.7%	29.0%	8.6%	
Sex (%)				0.013 *
Men	29.2%	12.9%	37.9%	
Women	70.8%	87.1%	62.1%	
Educational level (%)				0.035 *
University	22.5%	9.7%	29.3%	
Up to high school	77.5%	90.3%	70.7%	
Marital status (%)				0.001 **
Married	67.4%	45.2%	79.3%	
Not married	32.6%	54.8%	20.7%	
Employment (%)				0.649
Full-time job	2.2%	3.2%	1.7%	
Not full-time	97.8%	96.8%	98.3%	
Living status (%)				0.345
Living with others	91.0%	87.1%	93.1%	
Living alone	9.0%	12.9%	6.9%	
Depression (%)				0.032 *
Yes	12.4%	22.6%	6.9%	
No	87.6%	77.4%	93.1%	
Hypertension (%)				0.915
Yes	42.7%	41.9%	43.1%	
No	57.3%	58.1%	56.9%	
Diabetes (%)				0.805
Yes	18.0%	19.4%	17.2%	
No	82.0%	80.6%	82.8%	
Hyperlipidemia (%)				0.857
Yes	27.0%	25.8%	27.6%	
No	73.0%	74.2%	72.4%	
Alcohol				0.868
Yes	9.0%	9.7%	8.6%	
No	91.0%	90.3%	91.4%	
Smoking				0.419
Yes	6.7%	9.7%	5.2%	
No	93.3	90.3%	94.8%	
Sedentary Time (M±SD)	10.07 ± 1.26	10.51 ± 1.48	9.84 ± 1.07	0.602
<9 h/day	19.1%	16.1%	20.7%	
≥9 h/day	80.9%	83.9%	79.3%i	
Health Intake				0.497
Yes	69.7%	74.2%	67.2%	
No	30.3%	25.8%	32.8%	
BMI (kg/m^2^) M ± SD	24.29 ± 3.26	25.00 ± 3.72	23.92 ± 2.95	0.443
Normal weight (%)	53.9%	48.4%	56.9%	
Overweight (%)	46.1%	51.6%	43.1%	

BMI, body mass index (kg/m^2^); MVPA, moderate-to-vigorous physical activity; SD, standard deviation. * *p* < 0.05; ** *p* < 0.001.

**Table 2:** Descriptive statistics for objectively assessing physical activity and physical function.

		M (SD) (*n* = 89)
**Accelerometer variables**		
Wear time (min/day)		921.86 (84.99)
Daily MVPA time (min/day)		25.92 (22.63)
Daily Sedentary time (min/day)		604.34 (75.46)
**Muscle Strength**		
Handgrip Strength (Kg)	Baseline	25.14 (7.12)
	Follow-Up	26.31 (7.04)
Five-Times STS Test (s)	Baseline	6.86 (1.59)
	Follow-Up	6.78 (1.83)
**Balance**		
One Leg Standing Test (s)	Baseline	39.05 (22.55)
	Follow-Up	45.65 (21.85)

M, mean; MVPA, moderate-to-vigorous physical activity; *n*, number; SD, standard deviation.

**Table 3:** The percentage of balance and muscle strength change (improvement/maintenance or decline) between baseline and 12 months follow-up.

	Total (%)		MVPA < 15 min/day (%)		MVPA ≥ 15 min/day (%)	
	Improvement Maintenance	Decline	Improvement Maintenance	Decline	Improvement Maintenance	Decline
Grip strength	66.3	33.7	67.7	32.3	65.5	34.5
Five-Times STS	52.8	47.2	29.0	71.0	65.5	34.5
One Leg Standing	76.4	23.6	54.8	45.2	87.9	12.1

Five-times STS, five-times sit-to-stand test; MVPA, moderate-to-vigorous physical activity. Maintenance/improvement category, the value between baseline and follow-up is zero or positive. Decline category, the value between baseline and follow-up is negative.

**Table 4:** The association between 15 min MVPA per day and subsequent muscle strength and balance in older adults.

	Upper Strength		Lower Strength		Balance	
	OR (95% CI)	*p*-Value	OR (95% CI)	*p*-Value	OR (95% CI)	*p*-Value
**Model 1**						
**<15 min MVPA per day**	1.00 (ref.)		1.00 (ref.)		1.00 (ref.)	
**≥15 min MVPA per day**	0.905 (0.358–2.287)	0.833	4.644 (1.804–11.959)	0.001 *	6.000 (2.078–17.325)	0.001 *
**Model 2**						
**<15 min MVPA per day**	1.00 (ref.)		1.00 (ref.)		1.00 (ref.)	
**≥15 min of MVPA per day**	0.731 (0.210–2.542)	0.622	2.471 (0.767–7.957)	0.130	6.462 (1.455–28.704)	0.014 *
**Model 3**						
**<15 min of MVPA per day**	1.00 (ref.)		1.00 (ref.)		1.00 (ref.)	
**≥15 min of MVPA per day**	0.709 (0.202–2.488)	0.592	2.814 (0.825–9.596)	0.098	8.123 (1.611–40.966)	0.011 *

CI, confidence interval; MVPA, moderate-to-vigorous physical activity; OR, odds ratio. * *p* < 0.05. Model 1: Unadjusted model. Model 2: Adjusted for sociodemographic variables (age group, sex, educational level, living status, marital status, depression, hypertension, diabetes, hyperlipidemia, sedentary behavior, health intake, and body mass index). Model 3: Adjusted for sociodemographic variables, sedentary behavior, and accelerometer wearing time.

## Data Availability

The data used in this study are available from the corresponding author upon reasonable request.
